# Estimation of Trabecular Bone Volume with Dual-Echo Ultrashort Echo Time (UTE) Magnetic Resonance Imaging (MRI) Significantly Correlates with High-Resolution Computed Tomography (CT)

**DOI:** 10.3390/jimaging11020057

**Published:** 2025-02-13

**Authors:** Karen Y. Cheng, Dina Moazamian, Behnam Namiranian, Hamidreza Shaterian Mohammadi, Salem Alenezi, Christine B. Chung, Saeed Jerban

**Affiliations:** 1 Department of Radiology, University of California, La Jolla, San Diego, CA 92037, USA; 2 Research and Laboratories Sector, Saudi Food and Drug Authority, Riyadh 13513-7148, Saudi Arabia; 3 Department of Radiology, Veterans Affairs San Diego Healthcare System, La Jolla, San Diego, CA 92161, USA; 4 Research Service, Veterans Affairs San Diego Healthcare System, La Jolla, San Diego, CA 92161, USA

**Keywords:** quantitative MRI, ultrashort echo time, trabecular bone, fracture risk assessment, micro-computed tomography

## Abstract

Trabecular bone architecture has important implications for the mechanical strength of bone. Trabecular elements appear as signal void when imaged utilizing conventional magnetic resonance imaging (MRI) sequences. Ultrashort echo time (UTE) MRI can acquire high signal from trabecular bone, allowing for quantitative evaluation. However, the trabecular morphology is often disturbed in UTE-MRI due to chemical shift artifacts caused by the presence of fat in marrow. This study aimed to evaluate a UTE-MRI technique to estimate the trabecular bone volume fraction (BVTV) without requiring trabecular-level morphological assessment. A total of six cadaveric distal tibial diaphyseal trabecular bone cubes were scanned using a dual-echo UTE Cones sequence (TE = 0.03 and 2.2 ms) on a clinical 3T MRI scanner and on a micro-computed tomography (μCT) scanner. The BVTV was calculated from 10 consecutive slices on both the MR and μCT images. BVTV calculated from the MR images showed strongly significant correlation with the BVTV determined from μCT images (R = 0.84, *p* < 0.01), suggesting that UTE-MRI is a feasible technique for the assessment of trabecular bone microarchitecture. This would allow for the non-invasive assessment of information regarding bone strength, and UTE-MRI may potentially serve as a novel tool for assessment of fracture risk.

## 1. Introduction

Fragility fractures are highly prevalent and result in a significant financial cost and reduction in an individual’s quality of life. These fractures will likely increase in incidence with the growing population of older adults, with a predicted worldwide incidence of 6.3 million fragility fractures each year by 2050 [1]. Osteoporosis represents the most important treatable factor for these fractures. Bone mineral density (BMD), as measured by dual-energy X-ray absorptiometry (DXA) at the spine or hip, is the standard clinical measure to diagnose osteoporosis and estimate bone fracture risk [2]. Despite the widespread use of BMD in clinical practice, a diagnosis of osteoporosis (based on DXA T-score < −2.5) often fails to predict fracture risk accurately, with one study showing half of fractures occurring in individuals who do not meet the criteria for osteoporosis [3,4,5,6,7,8,9]. DXA does not distinguish between cortical and trabecular bone, and cannot quantify the microstructural properties of bone [9].

Osteoporosis is often considered a disease of trabecular bone, and both the number of trabecular elements and trabecular thickness have been shown to decline with age [9,10,11,12]. By histomorphometry, trabecular bone microarchitecture is quantified utilizing parameters such as the bone volume fraction (bone volume to total volume ratio, BVTV), trabecular number, trabecular thickness, and trabecular separation [13]. These same parameters have been applied to assessment of bone specimens by micro-computed tomography (μCT) [14], with the BVTV shown to be the strongest predictor of the mechanical properties of bone [15,16,17]. Although BMD and BVTV typically parallel each other, there are some diseases in which one changes while the other does not. For example, in osteomalacia, mineral content is reduced while osteoid volume stays the same [18]. In this case, BMD would be expected to decrease while BVTV would not. Since trabecular bone is more sensitive to changes related to osteoporosis and treatment of osteoporosis than cortical bone, assessment of BVTV could potentially yield important information regarding fracture risk and prevention that is currently not captured by DXA. To this end, BVTV as assessed by μCT has previously been shown to increase following bisphosphonate treatment [19]. However, μCT requires the acquisition of a bone specimen, which would be undesirable for routine bone quality assessment for fracture risk determination. As a result, there have been efforts to reproduce these measures with non-invasive in vivo imaging techniques such as high-resolution peripheral quantitative CT and MRI [14,20,21,22,23,24,25].

An increasing number of musculoskeletal research groups are investigating the potential benefits of utilizing magnetic resonance imaging (MRI) for bone evaluation [26,27,28,29,30,31,32]. MRI-based bone evaluation avoids the potential harmful exposures to ionizing radiation associated with X-ray-based techniques [25,33,34,35] and allows simultaneous assessment of the surrounding soft tissues [36,37].

Although conventional clinical MRI sequences can be used for morphological imaging, they cannot quantitatively evaluate bone due to the lack of detectable signal [26,27,28]. Specifically, the detected MR signal intensity of bone depends on several factors, including its apparent transverse relaxation time (T2*), which is very short [33,34] and cannot be captured by conventional clinical sequences. The T2* of bone is on the order of hundreds of microseconds, while the echo times (TEs) in conventional clinical MRI sequences are typically several milliseconds or longer [33,38]. As a result, previous attempts to evaluate trabecular bone by MRI have measured the degree of attenuation of signal from fatty bone marrow due the presence of bone rather than measuring the signal from bone directly [18]. In contrast to conventional clinical MRI sequences, ultrashort echo time (UTE) MRI sequences have the advantage of TEs on the order of several to tens of microseconds, enabling detection of signal from tissues with short T2* like bone [26,27,33,34,39,40].

UTE-MRI is underutilized in the evaluation of bone in part due to the high cost and time demands. Several research studies have focused on developing rapid and efficient UTE-MRI-based bone evaluation methods to facilitate clinical translational imaging of cortical bone [41,42,43,44,45,46,47]. To the authors’ knowledge, UTE-MRI has not yet been applied to the evaluation of trabecular bone. Imaging of trabecular bone with MRI is challenging due to lower image contrast, as trabecular features are on the order of 80–100 μm, beyond the resolution of conventional imaging methods [14,48]. In addition, evaluation of trabecular bone is challenging as the presence of fat in marrow results in chemical shift artifact that disturbs trabecular morphology [27,28]. The technique proposed in this study utilizes the signal from a UTE acquisition without the need for detailed trabecular-level morphologic assessment.

A UTE-MRI acquisition in trabecular bone likely represents the total detectable signal from bone (mostly bound water as limited intra-trabecular porosity is expected) and marrow (free water and fat). A similar acquisition at a TE around 2.2 ms likely represents detectable signal from free water and fat in the marrow, because bone signal has decayed to near zero values. In such an acquisition at 3T, free water and fat signals are in phase and there is no signal cancelation between them. Therefore, it can be hypothesized that the signal difference between UTE and TE = 2.2 ms divided by the UTE signal represents the bone volume to total volume ratio (BVTV) in trabecular bone sites.

This study investigates the feasibility of using dual-echo UTE MRI (TEs ≈ 0 and 2.2 ms) as a rapid technique for BVTV estimation in trabecular bone.

## 2. Materials and Methods

### 2.1. Sample Preparation

Fresh-frozen cadaveric lower-leg specimens from six donors (75 ± 4 years old) were provided by the UC San Diego School of Medicine Medical Education/Anatomical Services. Axial sections of the distal tibia, near the ankle joint, were cut into ~20 mm segments using a commercial band saw (B16, Butcher Boy, Selmer, TN, USA) in the frozen state. One 20 mm^3^ cube was excised from the metaphyseal region of each specimen using a low-speed diamond saw (Isomet 1000, Buehler, Lake Bluff, IL, USA). Only trabecular bone was included in the final bone cubes. After thawing, trabecular bone cubes were placed in a rectangular plastic container filled with perfluoropolyether (Fomblin, Ausimont, Thorofare, NJ, USA) to minimize dehydration and susceptibility artifacts during the MRI scans.

### 2.2. UTE-MRI and Data Analysis

The UTE-MRI scans were performed on a 3T clinical scanner (GE Healthcare, Waukesha, WI, USA) using an eight-channel transmit and receive knee coil. A dual-echo UTE Cones sequence with repetition time (TR) = 12.1 ms and TEs = 0.032 and 2.2 ms was performed. Images were acquired in the axial anatomical plane of the specimens, which was the standard coronal plane of the scanner. Field-of-view, acquisition matrix, slice thickness, voxel size, number of slices, and scan time were 100 mm, 200 × 200, 0.5 mm, 0.5 × 0.5 × 0.5 mm^3^, 40, and 5 min, respectively.

MRI images were analyzed in 10 slices in the middle of each specimen to avoid the artifacts on the upper and lower edges of the specimens caused by infiltrated air into the intertrabecular space. An experienced image analyst selected global regions of interest covering each specimen separately while avoiding edges.

Equation (1) was used to calculate the BVTV_Dual-Echo MR_ map in selected ROIs. The mean BVTV_Dual-Echo MR_ was calculated within each ROI to be compared with the µCT results.

(1) $${BVTV}_{Dual–Echo\;MR}=\frac{UTE\;signal-2nd\;Echo\;Signal\;(TE=2.2\;ms)}{UTE\;signal}$$

To investigate the feasibility of generating the BVTV_Dual-Echo MR_ map in vivo, the ankle of a healthy 24-year-old male volunteer was also scanned in the axial plane. The institutional review board (IRB) of the University of California, San Diego, approved this study. Written informed consent was obtained from the subject prior to participation. A clinical T1-weighted spin echo sequence was performed (TR = 788 ms, Flip angle (FA) = 142, matrix 352 × 352, FOV = 120 mm, in plane pixel size = 0.34 mm). A dual-echo UTE cones sequence with TR = 80 ms, and TEs = 0.032 and 2.2 ms, was also performed to generate the BVTV map. FOV, acquisition matrix, slice thickness, FA, and in plane pixel size were 120 mm, 160 × 160, 5 mm, 45, and 0.75 mm, respectively. BVTV_Dual-Echo MR_ was calculated for the distal tibia as described above.

### 2.3. µCT Imaging and Data Analysis

Specimens were also scanned using a GE eXplore 120 Preclinical µCT scanner (GE Healthcare, Waukesha, WI, USA) at 50 µm^3^ isotropic voxel size. Specimens were scanned in the same plastic container after emptying the liquid, as the liquid could downgrade the image contrast. Other scanning parameters were as follows: 100 × 100 × 165 mm^3^ FOV, 60 kV voltage, 32 mA current, 0.5° rotation step, and 100 min scan time.

Corresponding µCT images were selected manually (10 consecutive slices per each MRI slice). A 2D semiautomatic registration algorithm was used to map the selected ROIs onto the µCT images. Registration was performed by selecting matching corners of each specimen on MRI and µCT images using the MATLAB image processing toolbox (version 2021, The Mathworks Inc., Natick, MA, USA). Notably, a 3D automatic registration algorithm could not be applied due to the artifacts in MRI images caused by the trapped air in the intertrabecular space, particularly on the upper and lower edges of specimens. A local adaptive gray level thresholding algorithm (approximately 3 mm sub window dimension) was used to segment the bone pixels from the marrow pixels within each selected ROI on the MRI and µCT images. Equation (2) was used to calculate the mean µCT-based BVTV within each selected ROI.

(2) $${BVTV}_{\mu CT}=\frac{Bone\;voxel\;count}{Total\;voxel\;count}$$

### 2.4. Statistical Analysis

The measured BVTVs were not normally distributed as examined with the one-sample Kolmogorov–Smirnov test. A two-sided Wilcoxon rank sum test was used to examine the difference between MRI and µCT-based results. Spearman’s rank correlation coefficient with 95% confidence interval (CI) was calculated between MRI-based and µCT-based BVTV. The provided range of correlation coefficients was calculated with 1000 bootstrapping iterations, which considers for potential inter-specimen dependency between data points. *p*-values below 0.05 were considered significant. Statistical analysis was performed using SPSS software (version 29.0.2.0, IBM, Armonk, NY, USA).

## 3. Results

### 3.1. UTE-MRI Assessment of BVTV

**Figure 1:**
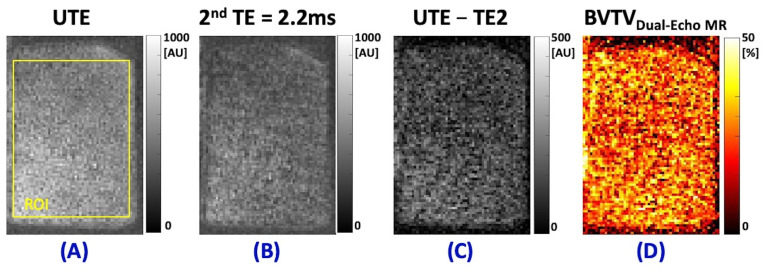
(**A**) UTE-MRI (TE = 0.032 ms), (**B**) second echo MRI image at TE = 2.2 ms, (**C**) subtracted second TE signal from UTE signal (representing the decayed bone signal), and (**D**) BVTV_Dual-Echo MR_ map (calculated from Equation (1)) of a representative trabecular bone specimen (excised cube from metaphysis in distal tibia) in the axial plane. The schematic region of interest (ROI) is depicted as a yellow box in (**A**).

Figure 1A shows the UTE-MRI image (TE = 0.032 ms) of a representative trabecular bone specimen in the axial plane. The schematic ROI selected in one slice in the middle of the specimen is depicted as a yellow box. This represents the total detectable signal from bone and marrow. Figure 1B shows the second echo MRI image at TE = 2.2 ms of the same specimen, which represents the signal attributable to marrow as the bone signal has decayed to near zero values. The second echo MRI subtracted from the UTE image (representing the decayed bone signal) is shown in Figure 1C. The BVTV_Dual-Echo MR_ map, which graphically represents the ratio of the subtracted signal (Figure 1C) and the UTE signal (Figure 1A) for the sample is illustrated in Figure 1D.

### 3.2. µCT Assessment of BVTV

**Figure 2:**
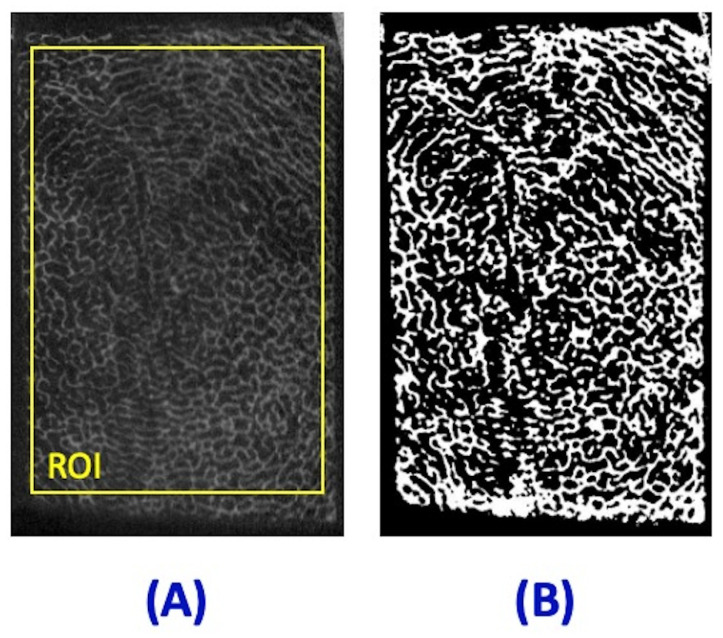
(**A**) μCT image of a representative trabecular bone specimen (shown in Figure 1) at 50 µm^3^ isotropic voxel size. (**B**) The segmented bone volume of the same specimen. The yellow box in (**A**) profiles the schematic ROI used for mean BVTV calculation.

The µCT image at 50 µm^3^ isotropic voxel size of the same representative specimen is shown in Figure 2A, in a matched slice with the MRI images shown in Figure 1. The schematic ROI selected in one slice in the middle of the specimen is depicted as a yellow box. The segmented bone volume for the same slice is shown in Figure 2B. In contrast to the MRI images in Figure 1, trabecular bone structure is obvious in the µCT images.

### 3.3. Comparison of BVTV_Dual-Echo MR_ and BVTV_µCT_

**Figure 3:**
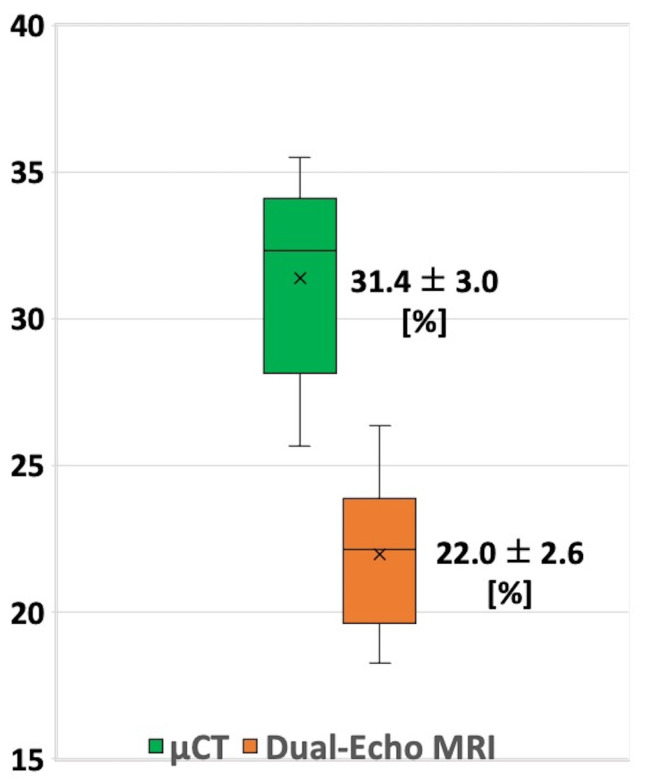
Box plots of BVTV_Dual-Echo MR_ and BVTV_µCT_ in scanned specimens. Mean, SD, first, and third quartile values are indicated in the box plots. MRI-based results were significantly lower than µCT (*p* < 0.01, two-sided Wilcoxon rank sum test).

Figure 3 shows box plots of the BVTV_Dual-Echo MR_ and BVTV_µCT_ results for all 10 slices evaluated in each of the six specimens and their mean values. The BVTV_Dual-Echo MR_ were significantly lower than BVTV_µCT_ (22.0 ± 2.6 versus 31.4 ± 3%, *p* < 0.01, two-sided Wilcoxon rank sum test). The difference between BVTV_Dual-Echo MR_ and BVTV_μCT_ for all measured regions was 9.4 ± 1.6% [5.2–12.2%] (range). The residual values from the linear fitting line were 0.0 ± 1.6% [−4.0–2.8%] (range).

**Figure 4:**
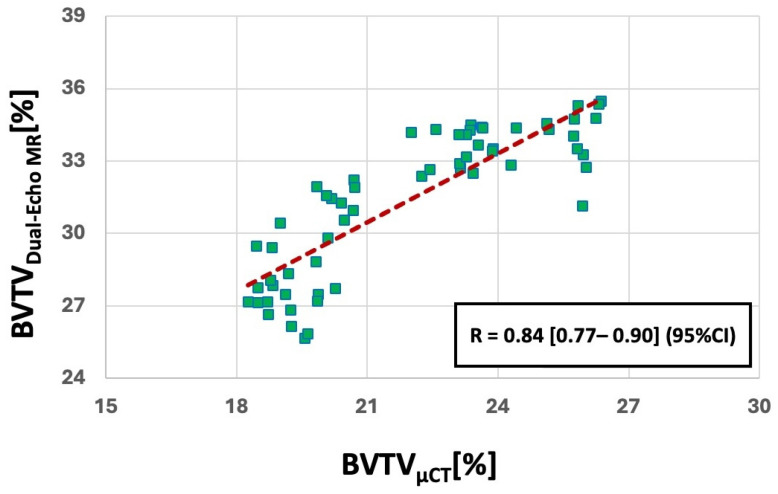
Scatter plots and linear trendlines of MRI-based BVTV on the µCT-based results. R value is Spearman’s correlation coefficient.

Figure 4 demonstrates the scatter plot and the linear regression of BVTV_Dual-Echo MR_ on BVTV_µCT_ for all the investigated ROIs. BVTV_Dual-Echo MR_ showed a strongly significant correlation with the BVTV_µCT_, with a Spearman’s correlation coefficient of 0.84 [0.77–0.90] (95% CI).

### 3.4. UTE-MRI Assessment of BVTV In Vivo

**Figure 5:**
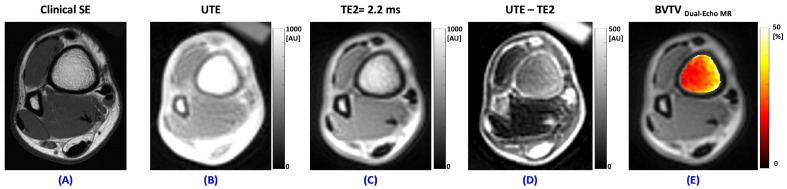
(**A**) Clinical axial T1 weighted spin echo sequence (TR = 788 ms, TE = 8.31 ms), (**B**), UTE-MRI (TE = 0.032 ms), (**C**) second echo MRI image at TE = 2.2 ms, (**D**) subtracted second TE signal from the UTE signal (representing the decayed bone signal), and (**E**) BVTV_Dual-Echo MR_ map (calculated from Equation (1)) of the distal tibia in a 24-year-old healthy male volunteer in the axial plane.

Figure 5A shows the clinical T1-weighted MR image of the ankle in a healthy 24-year-old man in the axial plane in vivo, with a greater number of trabeculae noted along the medial aspect of the distal tibia. Figure 5B shows the UTE-MRI image (TE = 0.032 ms) at the same level of the ankle in the axial plane, which represents the signal from both bone and marrow. Figure 5C shows an image through the ankle at the same level in the axial plane using the second echo MRI image at TE = 2.2 ms, which represents the signal from marrow after the bone signal has decayed to near zero values. Figure 5D shows the signal from the second MRI image subtracted from the UTE-MRI image, which represents the bone signal. The BVTV_Dual-Echo MR_ map for the distal tibia at this level is illustrated in Figure 5E. The color map illustrates the higher BVTV_Dual-Echo MR_ in the region where there is a greater number of trabeculae evident in the anatomic image along the medial aspect of the tibia.

## 4. Discussion

This study was the first to investigate the feasibility of using dual-echo UTE MRI as a rapid technique for BVTV estimation in trabecular bone sites. MRI-based BVTV showed a strong correlation with µCT-based results as the ground truth. The strong correlation, the non-invasive nature of MRI without ionizing radiation, the rapid acquisition time of the proposed technique, and the feasibility of applying this imaging technique in vivo, make it a promising tool for diagnosis and treatment monitoring of bone disease to prevent fragility fractures.

The current study expands on the existing literature utilizing MRI to evaluate trabecular bone structure by applying an MRI technique that enables direct detection of signal from the trabecular bone elements. Previous studies have evaluated indices of trabecular bone structure such as BVTV, trabecular thickness, and trabecular number, as well as trabecular BMD, and have shown these MRI measures to be correlated with DXA-based BMD values [18,23,49], as well as μCT-derived trabecular architectural measures [21,22] and biomechanical properties [22,50,51]. However, these studies utilized conventional MRI sequences in which the trabecular network is imaged as signal void. In such cases, an inverted image is needed to provide a value for signal intensity of the trabecular bone [22,49], the image is binarized into bone and bone marrow phases by thresholding [21,23,50], or the trabecular bone volume fraction is estimated by subtracting the marrow volume fraction [18]. Recently, the UTE-MRI acquisition on the fat-peak frequency resonance has been proposed to provide more accurate trabecular bone imaging and thickness calculation [52]. Nevertheless, these methods are limited by spatial resolution, which can produce partial volume effects that result in an apparent increase in BVTV, and may be dependent on the thresholding criterion utilized to binarize the bone and bone marrow phases [49]. In contrast, the dual-echo UTE MRI technique utilized in the current study directly measures the signal from both the trabecular bone and marrow such that neither high spatial resolution nor the use of a thresholding criterion is required.

In fact, in this study, the BVTV_Dual-Echo MR_, while highly correlated with the BVTV_μCT_, was consistently lower (rather than higher) than the BVTV_μCT_. We speculate that this may be related to the higher proton density in a volume unit of fat in marrow compared with that in water on average, which also results in an overestimation of the fat volume and total volume which, in turn, could contribute to the underestimation of BVTV in MRI. Future studies are needed to determine if the correlation between BVTV_Dual-Echo MR_ and BVTV_μCT_ is affected by the marrow fat content, which could be quantified by histology or alternative imaging methods such as iterative decomposition of water and fat with echo asymmetry and least squares estimation (IDEAL) or magnetic resonance spectroscopy (MRS) [53,54]. Notably, this systematic underestimation may vary for different anatomical regions and marrow compositions and may imply a non-linear relationship rather than a simple linear relationship.

The imaging technique introduced in this study was performed on a clinical scanner and could easily be performed utilizing a different imaging system. The echo times for the imaging sequence should not be modified as the first echo time in theory detects the total signal from bone and the second echo detects the signal after the bone signal has decayed; both are needed for the calculation of BVTV. However, other parameters such as repetition time and flip angle can be optimized depending on the resolution and field of view settings. These parameters may affect the estimated BVTV. Future investigation and potentially standardization may, therefore, be required before widespread use of this imaging technique.

This study does have several limitations. First, the studied ex vivo bone specimens were separated from overlying cortical bone and adjacent soft tissues. While we were able to perform the MRI technique and BVTV_Dual-Echo MR_ measures in vivo in a single volunteer, future in vivo investigation with greater numbers of participants will be needed to examine the reliability and reproducibility of the proposed techniques and to determine whether imaging the surrounding tissues will affect the calculation of BVTV_Dual-Echo MR_. Second, the studied trabecular bone specimens were harvested from distal tibias, whereas most fragility fractures occur in the proximal femora, spine, and forearms. A follow-up study would be needed to determine whether the same technique can be applied in bone more susceptible for fracture, and whether there are any significant differences in trabecular architecture between these regions. Third, while there is correlation between BVTV_Dual-Echo MR_ and BVTV_μCT_, and BVTV as measured by CT and histomorphometry has been shown to be the best determinant for the elastic properties of bone [15], as well as to increase following treatment of osteoporosis with bisphosphonates [19], it is not known whether BVTV_Dual-Echo MR_ can predict fracture risk. A longitudinal study may be needed to determine whether this MR measurement can predict fracture risk with equal or greater sensitivity than conventional DXA alone. Fourth, the impact of the noise level in the UTE-MRI images on the estimated BVTV values has not been investigated in this study and will need to be considered in future investigations. Finally, at most institutions, DXA represents a low-cost and widely available imaging technique. While the operational costs of MR remain a consideration, the rapid acquisition time achievable with this imaging technique affords an opportunity for addition of this sequence in the assessment of at-risk patients undergoing MRI for other indications, more so than the existing conventional MRI techniques for assessment of trabecular bone structure, which can have acquisition times of up to 15 to 73 min [22,49,50]. Additionally, our future quantitative MRI-based bone assessment may be further streamlined with the use of deep learning techniques [55,56,57,58], which can be applied to automate image segmentation and denoising.

## 5. Conclusions

Dual-Echo UTE MRI was investigated for its capability to determine the BVTV of trabecular bone in an ex vivo study performed on specimens from human distal tibial diaphyses. BVTV obtained by UTE-MRI showed significant correlations with BVTV determined by a μCT reference standard. The same imaging technique was applied to imaging a human volunteer and demonstrated the feasibility of determining BVTV_Dual-Echo MR_ in vivo. This study highlighted the UTE-MRI technique as a useful method to assess trabecular bone microstructural properties, which may be useful in future clinical studies for non-invasive and radiation-free fracture risk estimation.

## Data Availability

The data sets used and/or analyzed during the current study are available from the corresponding author on reasonable request.

## Abbreviations

The following abbreviations are used in this manuscript:

UTE	Ultrashort echo time
MRI	Magnetic resonance imaging
μCT	Micro computed tomography
BVTV	Trabecular bone volume fraction
DXA	Dual-energy X-ray absorptiometry
